# Somatosensory-guided tool use modifies arm representation for action

**DOI:** 10.1038/s41598-019-41928-1

**Published:** 2019-04-02

**Authors:** M. Martel, L. Cardinali, G. Bertonati, C. Jouffrais, L. Finos, A. Farnè, A. C. Roy

**Affiliations:** 1 Laboratoire Dynamique du Langage, CNRS UMR 5596 University Lyon 2, Lyon, France; 2 University of Lyon, Lyon, France; 3 The Brain and Mind Institute, Western University, London, ON Canada; 4 ImpAct Team, CRNL INSERM U1028, CNRS UMR5292, Lyon Neuroscience Research Center, University UCBL Lyon 1, Lyon, France; 5 Center for Mind/Brain Sciences (CIMeC), University of Trento, Rovereto, Italy; 6 IRIT, CNRS, Toulouse, France; 7 IPAL, CNRS, Singapore, Singapore; 8 Department of Developmental Psychology and Socialisation, University of Padova, Padova, Italy; 9 Hospices Civils de Lyon, Mouvement et Handicap & Neuro-immersion, Lyon, France

## Abstract

Tool-use changes both peripersonal space and body representations, with several effects being nowadays termed tool embodiment. Since somatosensation was typically accompanied by vision in most previous tool use studies, whether somatosensation alone is sufficient for tool embodiment remains unknown. Here we address this question via a task assessing arm length representation at an implicit level. Namely, we compared movement’s kinematics in blindfolded healthy participants when grasping an object before and after tool-use. Results showed longer latencies and smaller peaks in the arm transport component after tool-use, consistent with an increased length of arm representation. No changes were found in the hand grip component and correlations revealed similar kinematic signatures in naturally long-armed participants. Kinematics changes did not interact with target object position, further corroborating the finding that somatosensory-guided tool use may increase the represented size of the participants’ arm. Control experiments ruled out alternative interpretations based upon altered hand position sense. In addition, our findings indicate that tool-use effects are specific for the implicit level of arm representation, as no effect was observed on the explicit estimate of the forearm length. These findings demonstrate for the first time that somatosensation is sufficient for incorporating a tool that has never been seen, nor used before.

## Introduction

Over the last decades, several studies have contributed characterizing the phenomenon of tool embodiment following use. To date, many aspects have been described, making the theoretical framework of tool embodiment quite rich and articulated. Indeed, after the seminal work in monkeys by Iriki and collaborators^1^, it is nowadays widely accepted that tool-use leads to modifications in the multisensory peripersonal space in humans^2–4^. More recent work highlighted that tool use can also change body representations^5–8^. Numerous approaches, from perceptual to more action-related ones, have been pursued to assess tool incorporation into different body representations (see, for review^9^) among which the implicit body representation for action, often termed body schema in contraposition to body image, a more explicit and conscious body representation^10,11^.

Different senses contribute to tool embodiment: typically, multisensory integration is necessary for tool use effects to emerge when perception in the peripersonal space is assessed^7,12–16^. Instead, mere vision of tool-use is sufficient to modify either tactile distance perception on the body^17^, or tasks impinging on reachability estimates^18^. Here, we assessed the role of somatosensation for incorporation of tool into the implicit body representation for action, assessing the changes in movement kinematics as a proxy for body representation update.

Body representation for action is indeed conceived as a highly plastic representation reflecting the different changes our body undergoes: from the slow growth in size from birth to adulthood, to the sudden size changes produced by transient functional lengthening during tool manipulation. When we use tools to achieve a particular goal, our body size and structure change abruptly, which is thought to be reflected in the update of the implicit body representation^19^. For example, grasping with a mechanical grabber modifies, in few minutes, participants’ arm length representation^5^. In previous work, we asked participants to grasp and point to an object with their right hand before and after using a 40 cm-long mechanical grabber with the same right hand. The kinematics of both free-hand grasping and pointing movements performed after tool use was modified in a specific and selective way: only the arm-related component of the movement (called transport component) was modified, displaying longer latencies and smaller peaks in several movement parameters (e.g., velocity, deceleration), whereas no change was observed on the hand-related one (called grip component). Post tool use kinematics thus reveals that participants’ arm (but not hand) representation was modified and suggests this change reflects a longer arm representation. Arm represented length’s increase was indeed supported by comparing differences naturally present among long(er)- and short(er)-armed participants: people with longer arm similarly displayed longer latencies and smaller peaks in transport movement parameters. Convergent evidence from a touch localization task showed that wrist-elbow delivered touches (but not wrist-fingertips) were localized farther apart after use of the same arm-elongating grabber^5^ (see also^17^). Altogether, these findings support the old tenet that humans incorporate tools in the action related body representation^9,20^. Yet, since all the previous studies were performed by assessing visually guided tool use actions, the role played by somatosensation per se in tool incorporation remains elusive.

In principle, both vision and somatosensation could contribute to tool incorporation, and recent work has highlighted their integration^21^. While vision seems to be predominant for space and objects coding since infancy^22–24^, proprioceptive and tactile signals appear to be critical information for the update of this implicit action-related body representation. Since its seminal conceptualization, the body schema has been thought as mainly fed by proprioceptive signals^25^. By varying the participants’ hand felt or seen orientation during a classical visual hand laterality task^26^, Shenton and colleagues determined that proprioception indeed plays a dominant role for this implicit body representation^27^. In a recent single case study of a deafferented patient, we reported that preserved somatosensation is necessary for tool incorporation in the body representation for action to occur^28^. Importantly, healthy participants are able to accurately estimate the length and barycenter of unseen rods if allowed to wield them^29–31^, suggesting that the brain can form a correct representation of a held object based on somatosensory inputs alone.

However, whether somatosensation alone is sufficient for tool-use-dependent body representation plasticity remains unknown. Here we addressed this question by comparing movement’s kinematics in blindfolded healthy participants when grasping an object before and after tool use. Crucially, vision was prevented throughout the experiment, as participants had no visual information about the set-up, the tool they had to use, the object to be grasped or even the room they were performing in.

Based on the hypothesis that somatosensation is crucial for body schema plasticity^9,32^, we predicted to observe kinematic differences between pre and post blindfolded tool-use. We expected the transport component to be selectively affected, displaying a pattern similar to what found in previous studies where both vision and somatosensation were available in the same tool-use paradigm^5,33,34^, namely lower peak amplitude and longer peak latencies in transport but not in grasping parameters. Repeatedly bringing the hand to a given position in space is known to increase precision of position sense^35^. To control for possible spatially selective effects of tool-use on somatosensation, participants performed the free-hand grasping actions either in the same space (group 1) or in an orthogonally oriented space (group 2) with respect to the space and the direction in which they performed the tool use movements. Further, to test whether tool-use changes are produced also at an explicit level of body representation, we additionally used an explicit task of arm-length estimation^10^.

## Experiment 1

### Material and methods

#### Participants

We recruited forty-six participants without any known neurologic impairment (21 males; mean age ± SD: 23.1 ± 4.1; range from 18.8 to 32.3) and randomly assigned them to two groups of 23 participants (group 1: 11 males; mean age ± SD: 22.7 ± 1.2 years; range from 20.4 to 25.2, and group 2: 10 males; mean age ± SD: 23.4 ± 3.3; range from 18.8 to 32.3; *t*(44) = −0.90; *p* > 0.250). All participants were right-handed and received a monetary compensation for their participation. They all gave written informed consent to participate to the study, which was approved by the ethics committee (*Comité de Protection des Personnes* Sud Est IV) and conformed to the principles of the revised Declaration of Helsinki^36,37^.

#### Apparatus and procedures

**Figure 1:**
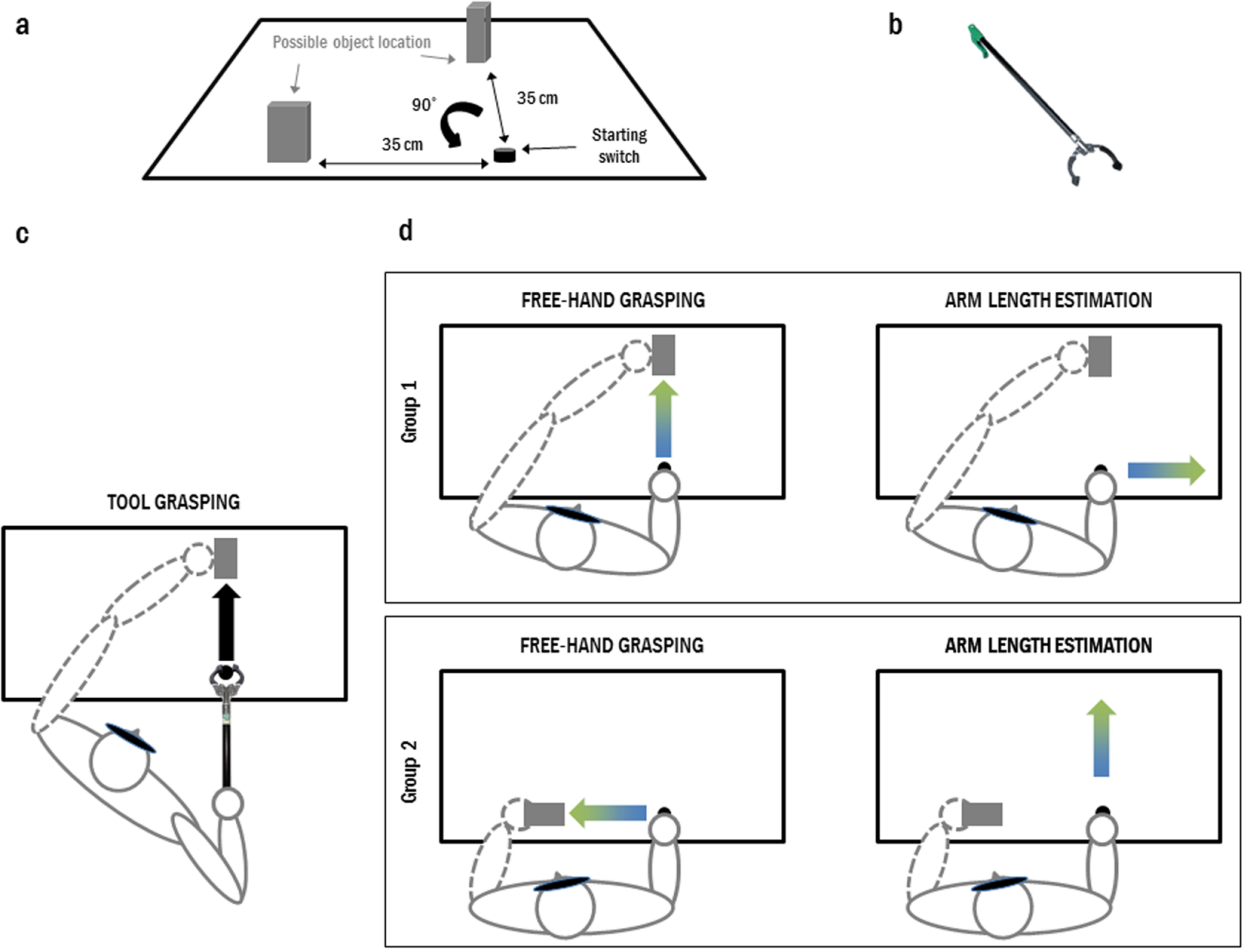
Experimental set-up. (**a**) A wooden object was placed on the table either in front or to the left of the hand starting position. In both cases, the distance between the starting switch and the object was 35 cm. (**b**) Picture of the mechanical grabber used in Experiment 1. (**c**) Tool use was always performed along a sagittal axis. (**d**) Left column: Before and after tool use, upon hearing a go signal, subjects had to reach and grasp the wooden target object with their hand: along the sagittal axis for Group 1, and along the frontal axis for Group 2. Right column: The Estimation task consisted in sliding their index finger for a distance matching the one between their elbow and wrist, along a frontal axis for Group 1, along the sagittal axis for Group 2.

Participants were first blindfolded and then, with the help of the experimenter, entered the experimental room. They were comfortably seated in front of a table, the right hand in a pinch-shaped grip (thumb and index fingertips touching) on a switch. Their left hand, flattened and palm down on the table, was kept in contact with the target object, a wooden block (10 * 2.5 * 5 cm, 96 g) placed on the table. The object was placed by the experimenter between the thumb and index fingertips, the corner of the wooden block contacting the skin of both the index and thumb (specifically, the lateral surface of the thumb and index fingertips). Participants were instructed to keep their hand flat, palm down on the table and keep gentle contact with the objet. Depending on which group the participant belonged to, the object was either lined up with participant’s right shoulder at 35 cm along the sagittal axis, or 35 cm along the frontal axis on the edge of the table (see Fig. 1a).

**Figure 2:**
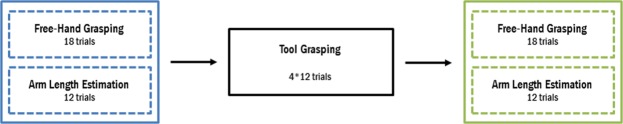
Experiment 1 timeline. Tool use session included 4*12 trials. Pre and post tool use sessions consisted of 18 free hand grasping movements and 12 arm length estimation trials. The two tasks were run by blocks and the order of the blocks was counterbalanced across participants.

Three sessions composed the experiment: a pre and post tool use session separated by a tool use session. The pre and post tool use sessions were identical and consisted of two tasks proposed in a counterbalanced order across participants (see Fig. 2): a free-hand grasping task (Implicit task; 18 trials), in which participants were required to reach, grasp and lift a target object with their right hand, and an arm length estimation task (Explicit task; 12 trials), where participants had to slide their right index finger on the surface of the table from the starting position to a final point for a distance they estimated to be equal to the distance between their wrist and elbow, which were named and touched by the experimenter while giving the tasks instructions to the participant. Participants were touched only once, at the beginning of each length estimation task session (pre and post), on the elbow and wrist of their right dominant arm, recalling them what was the anatomical distance to be estimated. Notably, this task involves no pointing or implicit knowledge of the body posture such as in the forearm bisection task^6,38^, typically used to investigate the body representation for action. It is a non-visual alternative metrics to measure the explicit knowledge of the arm length such as the line length task, where participants judge whether a line is equally long as different parts of their hand^39,40^. In both tasks, performance measures were derived from Ired markers displacement via an optoelectronic high-resolution motion tracking device (see below). In both tasks, once a trial performed, participants returned to the starting position (pinch grip position on the switch) and waited for an auditory ‘go’ signal to perform the next trial. The entire experiment was video-recorded. Actual forearm length was measured as the linear distance between the ulnar styloid process (at the wrist) and the humeral medial epicondyle (at the elbow) at the end of the experiment.

The tool use session consisted of four blocks of 12 trials each (48 trials in total). We instructed participants to place, at the beginning of each trial, the tip of the tool on the same starting switch used in the pre and post tool use phases and wait for the auditory go signal, upon which they had to use the tool to reach and grasp the same target object used in the free-hand grasping task. The tool was a commercial grabber (Unger Enterprise Inc, CT, USA; see Fig. 1b) with an ergonomic handle (9 cm) fitted with a lever, a 33-cm-long rigid shaft, and a “hand” with two articulated fingers (10 cm). Squeezing the lever (vertically) made the “fingers” of the tool closing (horizontally). Participants were not allowed any previous practice with the tool and had never used it or seen it before the day of testing, though they could haptically explore it with both hands before starting the experiment.

The pattern of changes in kinematics (lower peaks amplitudes and longer peaks latencies) expected here, has previously been proven to be specific of tool use, as it is absent in control situations whereby the tool-use task was replaced by an equal number of grasping movements performed both with the free hand (Baccarini *et al*.^33^; Experiment 2 of the present study) and with a weight -corresponding to that of the tool- attached to the hand^5^, or even when the same tool was wielded but not used^34^. While this previous evidence did not urge us to include a non-tool use condition in Experiment 1, it is important to consider that repetition of even relatively simple manual reaching movements towards a given position has been reported to enhance proprioception selectively at that location. Wong and colleagues^35^ found that proprioceptive acuity improved in the trained region of workspace, whereas no proprioceptive improvement was found when tested elsewhere. We deemed therefore relevant for the present study to control for the possible confound that kinematics changes may occur because of changes in proprioception, instead of the hypothesized changes in the representation of the arm length. To run such a control, both groups performed the tool use session towards the same frontally located object (see Fig. 1c), but while group 1 performed the free hand grasping (before and after tool use) towards the same position, group 2 acted laterally, toward an orthogonally oriented position (see Fig. 1a–d). As tool-use effects duration is not known, we opted for a between subject design to avoid carrying over effects on free hand movements. This design, by dissociating movement direction between pre/post tool use tasks and the tool use session, allows to make alternative predictions. If tool use modifies proprioception in a spatially selective manner, then kinematics should be altered only in the position where the tool was directed to (leading to an interaction group * Session: pre-post). Instead, if tool use modifies the represented arm length used to guide manual actions, then kinematics should be altered irrespective of position (leading to a main effect of Session).

We designed the control for the implicit nature of the effects similarly, distributing the explicit task orthogonally between the two groups. While all participants used the tool along the sagittal axis, participants in group 1 performed the implicit task along the same axis and the explicit task on the frontal axis (toward the right side). Participants in group 2 performed the implicit task along the frontal axis (toward their left) and the explicit task along the sagittal axis (see Fig. 1d).

#### Kinematic recording system

**Figure 3:**
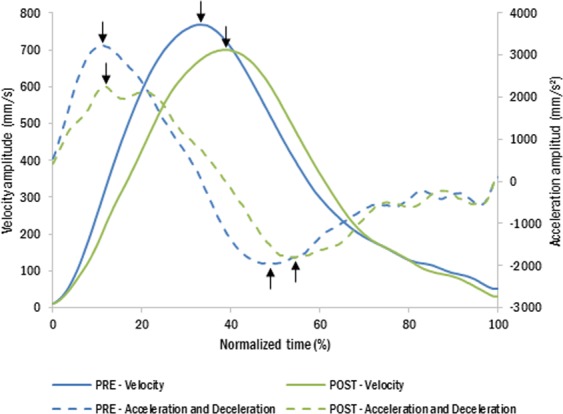
Kinematic profile of a representative subject (experiment 1; group 1) before (blue) and after tool use (green). After tool-use, the peaks were shorter and delayed. Black arrows indicate the positions of the extracted parameters, in amplitudes and latencies.

We placed three infrared light emitting diodes (IREDs) on the participants’ right hand: on the medial lower corner of the thumb nail, on the lateral lower corner of the index finger nail and on the skin proximal to the styloid process of the radius at the wrist. Three more IREDs were located on the tool: on its “fingers” and on the distal part of the shaft (“wrist”). The reaching component of the movement was derived from the wrist marker, and the grip component was derived from the thumb and index finger markers. Spatial localization of the markers was recorded with an Optotrak 3020 (Northern Digital Inc, Ontario, Canada; sampling rate: 200 Hz; 3D resolution: 0.01 mm at 2.25 m distance). For each free-hand grasping movement, we extracted and analyzed off-line the following parameters: latencies and amplitudes of the wrist acceleration, velocity and deceleration peaks (reaching component, Fig. 3), and latency and amplitude of the maximum thumb-index distance (hereafter MGA for Maximum Grip Aperture) (grasping component). For each movement we measured the overall movement time as the time between the beginning of the movement (velocity ≥ 10 mm/s after switch release) and stabilized grasp on the object (before object lifting). When participants accidently missed the object in free-hand reaches, we repeated the trial at the end of the session. For the arm length estimation task, we computed the distance between the starting switch and the end point of the index finger displacement using the IRED marker on the index finger. Participants’ right forearm actual length (from the wrist to the elbow) was measured and compared to the estimated length. Participants’ right arm (from the wrist to the shoulder) and forearm actual length (from the wrist to the elbow respectively) were measured. Arm length was measured on a different day (due to some participants’ unavailability, arm length was measured for 30 participants out of 41; 15 for each group). Data on forearm length was missing for 3 participants out of 41.

#### Statistical Analysis

Two participants who changed posture between the pre and post tool use sessions and three who exceeded (>2.5 standard deviation) population mean values in at least two kinematic parameters, were excluded from analyses. Additionally, two participants of group 1 did not correctly understand the length estimation task instructions (they estimated the elbow-middle fingertip distance instead of the elbow-wrist distance); their data for this task have been discarded from analysis. Hence, analyses were conducted on data from 22 participants from group 1 (11 males; mean age ± SD: 22.8 ± 1.2 years; range from 21.0 to 25.2) and 19 from group 2 (8 males; mean age ± SD: 23.6 ± 3.4 years; range from 18.8 to 32.3).

We performed a full factorial design permutation analysis^41,42^ on free-hand movement kinematic parameters (implicit task), using the flip package on R^43,44^. As the arm length estimation (explicit) task involved only one parameter, a repeated measures ANOVA was performed on data from such task. Both the full factorial design and the ANOVA were with Group as a between-subject factor and Session (pre/post) as a within-subject factor to evaluate (1) the effect of tool use session on body representations and (2) the presence of these effects across different movement directions. Although similar to an ANOVA, the interaction test in this permutation analysis differs in the calculation as it is the comparison of the differences between pre and post among the two (independent) groups. Importantly, the permutation analysis we applied in a previous study^45^ is designed for a multivariate framework; it allows to combine the significance of the kinematic parameters for the reaching phase (amplitudes and latencies of peaks the wrist acceleration, velocity and deceleration) and those for the grasping phase (amplitudes and latencies of Maximum Grip Aperture), to obtain one global p-value for each component. The global p-value is obtained via Nonparametric Combination (NPC^46^) of partial p-values testing the single parameters. The methodology accounts for dependence among tests through a nonparametric approach based on the joint (i.e. multivariate) permutation distribution. For interpretation purposes, the global test is analogous to a MANOVA approach, but with less assumptions on the data and better inferential properties^41,42^.

Moreover, for sake of comparison, kinematics without vision from group 1 was statistically compared to kinematics obtained under condition of full vision and somatosensation from Cardinali and collaborators^5^, as the two experiments used the same paradigm, movement direction and tool. Sensory modality (somatosensation only in this study: n = 22 vs. somatosensation and vision in Cardinali’s data: n = 14) was used as between-subject factor and Session (pre/post) as within subject factor.

Following our prediction that tool use increases the represented arm length, and previous kinematic differences observed between short(er) and long(er)-armed participants^34^, we additionally ran correlation analyses between participants reaching parameters of the pre session (i.e. before tool use) and their actual arm length (both arm and forearm). The motivation for this approach is that if tool use effects consist in lengthening the represented length of the arm, naturally longer armed participants should show -naturally- different transport kinematics with respect to shorter armed participants. Using the flip package, we performed one-tail Pearson correlations grouping the parameters from the reaching phase to obtain a global p value with Fisher combination. As data on arm length was not available for all, we performed these correlations on the data from 30/41 participants (for the arm) and 38/41 (for the forearm).

### Results

#### Implicit task

The permutation analysis revealed significant Group effects on the wrist velocity peak after Fisher combinations (group 1: 742 mm/s; group 2: 585 mm/s; *K* = 15.88, *p* < 0.001, η²_p_ = 0.30). Group 2 participants opened their fingers less than those of group 1 (group 1: 105 mm; group 2: 96 mm; *K* = 6.50, *p* = 0.028, η²_p_ = 0.12). Noteworthy, this permutation analysis revealed no interaction between Group and Session in any of the movement parameters (all p > 0.219), suggesting any effect of tool use was not limited to a given direction or portion of the workspace.

**Figure 4:**
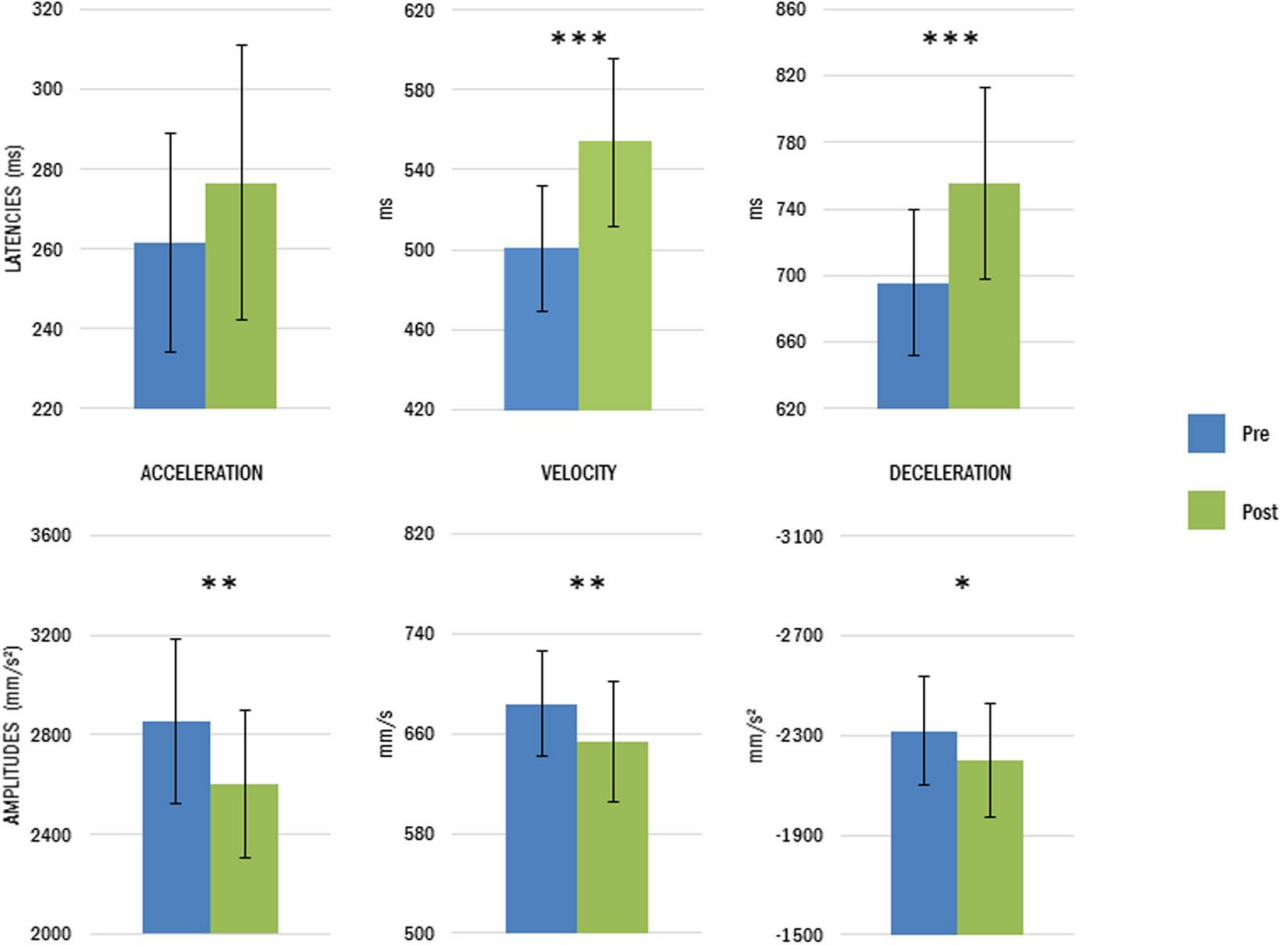
Tool use modifies movement kinematics of the reaching component. After tool use (green), participants (group average) showed longer latencies (upper graphs) and smaller peak amplitudes (lower graphs) compared to before tool use (blue). Asterisks denote significant differences between pre and post. *<0.05; **<0.01; ***<0.001. Bars illustrate mean values for each parameter and the 95% confidence interval.

Indeed, the analysis also revealed a main effect of Session on most of the transport parameters. Movements performed after tool use were characterized by a reaching phase with longer peak latency for both velocity and deceleration (velocity peak latency: 501 vs. 554 ms; *t* = 3.956, *p* < 0.001, η²_p_ = 0.28; deceleration peak latency: 695 vs. 755 ms; *t* = 3.216, *p* < 0.001, η²_p_ = 0.21). In addition, all the peaks amplitudes were reduced after tool use (acceleration peak amplitude: 2852 vs 2603 mm/s²; *t* = −3.17, *p* = 0.002, η²_p_ = 0.20; velocity peak amplitude: 684 vs 654 mm/s; *t* = −3.06, *p* = 0.004, η²_p_ = 0.20; deceleration peak amplitude: −2318 vs −2199 mm/s², *t* = 2.04; *p* = 0.047, η²_p_ = 0.10). Figures 3 and 4 illustrate these tool-induced changes. No significant effects were found on the movement time (*t* = 1.58; *p* = 0.121, η²_p_ = 0.06), or on the grip component of the movement (MGA latency: *t* = 1.44; *p* = 0.159, η²_p_ = 0.05; MGA peak: *t* = −1.74; *p* = 0.090, η²_p_ = 0.07).

A highly significant global p-value highlights that the kinematic changes were present for the transport component (Fisher combination; *K* = 32.94, *p* < 0.001), as the grasping component was not significantly affected by tool use (*K* = 4.23, *p* = 0.080). There was no interaction between the two groups, neither for the reaching phase nor for the grasping phase (all *p*s > 0.389), reinforcing the similar behavior after tool-use whatever the movement direction tested.

For the sake of precision, we also run the analysis on each group separately to verify if the significant difference was still present. On group 1, there was a significant effect of session on the amplitude of acceleration (*t* = −4.14; *p* < 0.001), latency of velocity (*t* = 4.26; *p* < 0.001), deceleration (*t* = 3.30; *p* = 0.003), and movement time (*t* = 2.01; *p* = 0.049). The global p-value indicated that kinematic changes were present only on the transport component (Fisher combination; *K* = 27.63, *p* = 0.001), but not on the grasping one (Fisher combination; *K* = 2.52, *p* = 0.278). On group 2, there was a significant effect on the velocity amplitude (*t* = −2.74; *p* = 0.03), and trends on the velocity latency (*t* = 1.75; *p* = 0.091), velocity deceleration (*t* = 1.79; *p* = 0.067) and deceleration amplitude (*t* = 1.74; *p* = 0.098). The overall p value was close to significance for the transport component (Fisher combination; *K* = 13.21, *p* = 0.058) but not for the grasping one (Fisher combination; *K* = 2.44, *p* = 0.281). Such results confirmed the global analysis performed earlier, with specificity of the kinematics effect on the transport component.

When comparing results from Group 1 of this study with those from Cardinali and collaborators^5^, we found a main effect of Session for each parameter from the transport component (all *ps* < 0.021). Participants reached the object with longer latencies and smaller amplitude after tool-use. We additionally observed a main effect of Sensory modality, as blindfolded participants (i.e., somatosensation alone) were slower on all kinematic parameter (all *p*s < 0.001) with respect to non-blindfolded participants (i.e., vision and somatosensation), except for the acceleration latency (Fisher combination; *K* = 4.54, *p* = 0.091). Permutation analyses also revealed a significant interaction between the factor Sensory modality and Session for the whole transport component (Fisher combination; *K* = 25.58, *p* < 0.001). Performing tool-use with both vision and somatosensation further decreased the amplitudes of the transport component parameters (acceleration: *t* = −3.253, *p* = 0.002; velocity: *t* = −4.057, *p* < 0.001; and deceleration: *t* = 4.633, *p* < 0.001). We found no interactions between Sensory modality and Session for any of the latencies (all ps > 0.428). When considering the grasping component, there was no main effect of Session, as tool-use did not affect either the latency, or the amplitude of MGA (all ps > 0.109), thus reinforcing the selectivity of kinematic changes for the transport component of movements. A main effect of Sensory modality indicated a larger and delayed MGA while grasping without vision (all ps < 0.001). We found no interaction between Sensory modality and Session for the grasping component (Fisher combination; *K* = 3.25, *p* = 0.168).

#### Explicit task

When comparing the arm length estimation before and after tool use, we found neither main effects, nor interaction, indicating that tool use did not affect the explicit representation of arm length. The estimated length was similar before and after tool use (pre: 255 mm; post: 251 mm; F(1, 37) = 0.86, *p* = 0.360). Participants forearm estimation corresponded to 94% and 92% of their actual forearm length before and after tool use, respectively.

#### Actual arm length correlations

**Figure 5:**
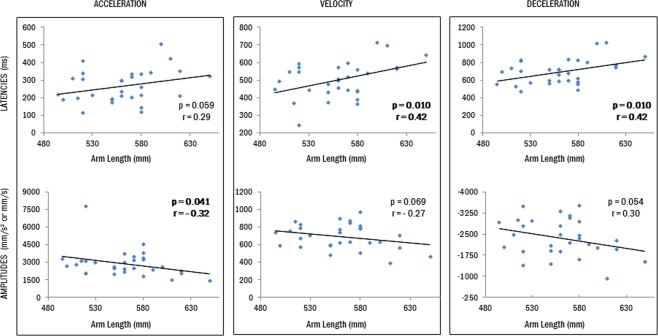
Correlation between kinematic parameters (peak latencies and amplitudes) and participants’ arm length. The graphs show the time at which each subject reached a particular peak (upper panels) and the amplitude of the peak (bottom panels) from the pre tool use session, plotted against the participants’ arm length. Each dot represents a subject (N = 30). Significant correlations are indicated in bold. The longer the participants’ arms, the longer their latencies and the lower the peaks amplitude.

Finally, we examined the correlation between participants’ arm length (ranging from 495 to 650 mm) or forearm length (ranging from 245 to 305 mm) and the 6 kinematic parameters of the transport component as measured before tool use. Significant correlation (see Fig. 5) indicated that participants having longer arms exhibited longer latencies than participants with shorter arms (velocity: *r*(28) = 0.42, *p* = 0.010; deceleration: *r*(28) = 0.42, *p* = 0.010; a similar but non-significant trend was observed for the acceleration peak latency *r*(28) = 0.29, *p* = 0.059). Regarding peaks amplitude, longer armed participants showed reduced acceleration (*r*(28) = −0.32, *p* = 0.041) and, marginally, deceleration (*r*(28) = 0.30, *p* = 0.054). A non-significant trend was visible for velocity (*r*(28) = −0.28, *p* = 0.069). The Fisher combination from the permutation approach indicated that the overall transport component was significantly correlated to participants’ arm length (*K* = 20.8, p = 0.018), confirming the tight relation between arm kinematic and morphology and in particular between the specific pattern of longer latencies and reduced peaks and longer arm length. As for the forearm, the same pattern was observed with reduced acceleration (*r*(36) = −0.33, *p* = 0.014) and deceleration (*r*(36) = 0.37, *p* = 0.012) amplitudes for participants with longer forearms. A similar non-significant trend was observed for the amplitude of the velocity (*r*(36) = −0.25, *p* = 0.063). Once again, the Fisher combination indicated that the overall transport component was significantly correlated to participants’ forearm length (*K* = 16.8, p = 0.041)

### Interim discussion

The results of Experiment 1 clearly favor the hypothesis that somatosensation is sufficient for the plastic changes of arm length representation to emerge. Control condition (between groups) and task (within groups) further show the effects are not limited to a given (trained) sector of space and emerge selectively at an implicit level (reaching kinematics), without affecting the explicit, subjective estimate of one’s own arm length.

Besides observing the predicted pattern of results on kinematics, discussed in detail below (Discussion section), the post tool use session also revealed a previously unreported phenomenon: 13 participants (out of 22) from group 1, and 4 participants (out of 19) from group 2 failed to grasp the object in the very first few trials (trial 1 to max 4, out of 18) because they would not reach far enough to come in contact with the object. In addition, some participants (group 1: 5/22 participants; group 2: 3/19 participants) sometimes missed the starting point on their way back to it at the end of the trial. Backward reaching movements were too long, bringing the hand between the starting point and the chest.

This misreaching behavior might be due to the arm-retracted posture during the tool-use session, which may have affected their felt starting position. To maintain the same distance between the effector (hand or tool) and the object throughout the experiment, participants were instructed to place the tip of the tool prongs on the same starting switch where they positioned their index and thumb fingertips during free-hand grasping. To do so, they had to move the scapula posteriorly and medially along the back (scapula retraction) resulting in a different arm posture. Changing the starting position is known to affect movements modifying the arm end posture^47^, inducing directional biases^48^, or altering kinematics^49^. These studies, however, focused on visually guided movements and did not investigate the effects potentially emerging when postural changes are only driven by proprioception. Noteworthy, Vindras and colleagues^50^ observed that initial hand position influences pointing errors when only proprioception is available, these effects disappearing when hand vision is also allowed. We thus reasoned that postural changes between free-hand and tool-mediated reaching (scapula retraction) may be responsible for dysmetric reaching, independently of the tool-induced kinematic changes reported above.

**Figure 6:**
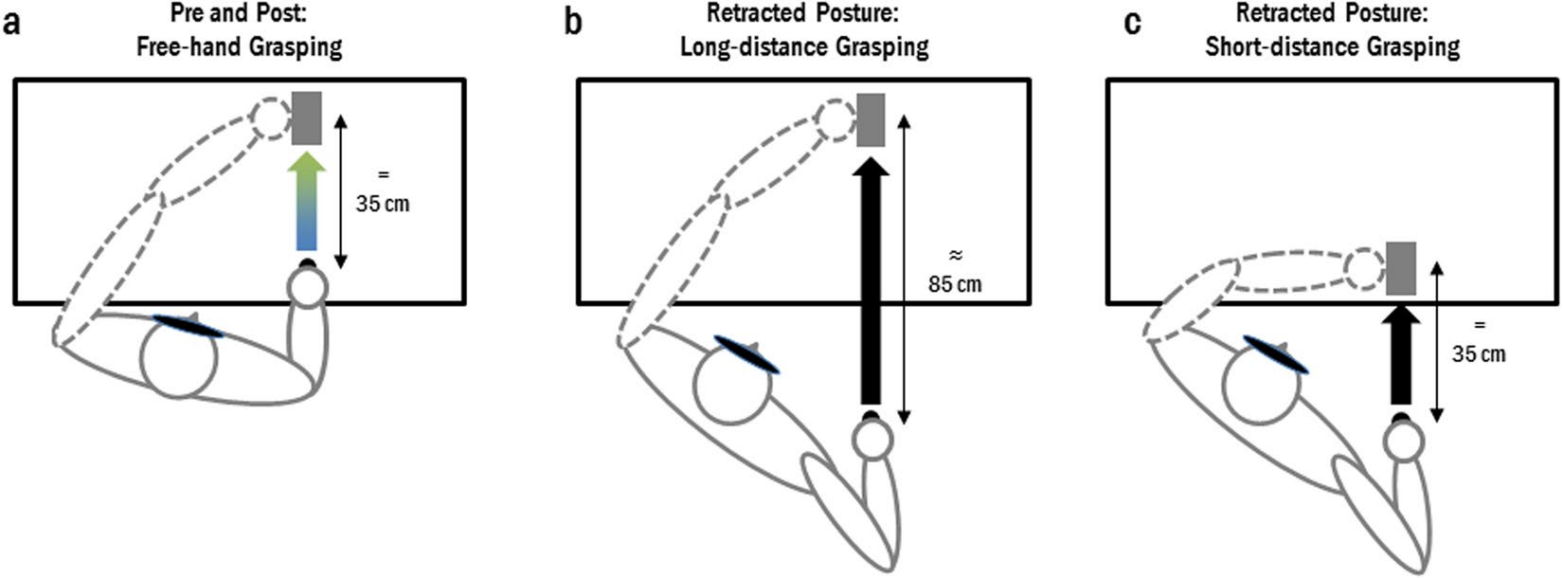
Experimental set-up for Experiment 2. (**a**) A wooden object was placed on the table in front of the starting position, at 35 cm. Before and after the two sessions with retracted starting posture, inducing either a long- (**b**) or short-distance (**c**) grasping movement, participants reached out and grasped the wooden target object with their right hand upon hearing a go signal. (**b**) In the Long-Distance session the initial posture was the same as the one assumed while using a 40cm-long grabber in Experiment 1, with the object to grasp at the same position than in the pre and post tool use sessions (e.g. about 85 cm from this new starting point). (**c**) In the “Short-Distance” session participants maintained the same initial posture as in the Long-Distance session while the target object was positioned 35 cm from the acting hand.

To test this hypothesis, we performed a second experiment using the same pre/post paradigm as in Experiment 1. Importantly, in Experiment 2 participants did not use a tool, but performed an equivalent number of free-hand movements adopting an initial arm position that mimicked the one adopted during tool use session of Experiment 1 (Scapular retraction; see Fig. 6). Before and after these retracted posture sessions, participants had to perform free-hand grasping movements as in Experiment 1, thus Experiment 2 also provided a further control for the specificity of the tool-use effects obtained in Experiment 1. We hypothesized that (1) modifying the initial position of the hand would be sufficient to induce dysmetric reaching in the post session, and (2) in the absence of tool use, we should no longer observe the kinematics changes that followed tool-use in Experiment 1.

## Experiment 2

### Material and methods

#### Participants

We recruited twenty participants without any known neurologic impairment (10 males; mean age ± SD: 22.3 ± 2.4; range from 18 to 26.6). None of them took part in the first experiment. All participants were right-handed and received a monetary compensation for their participation. They all gave written informed consent to participate to the study, which was approved by ethics committee (*Comité de Protection des Personnes* Sud Est IV) and conformed to the Helsinki declaration.

#### Apparatus and procedures

**Figure 7:**
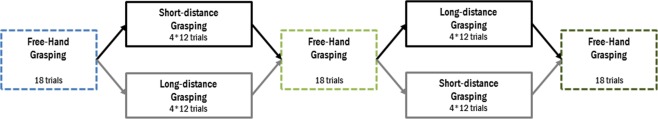
Experiment 2 timeline. Each participant performed two retracted arm posture sessions (Short-Distance and Long-Distance Grasping) preceded and followed by a block of free-hand grasping movements. The order of the two sessions was counterbalanced across participants.

Apparatus and procedure were the same as for Experiment 1 except that Experiment 2 was a within-subject design, composed of 5 sessions (see Fig. 7): one before and two after two retracted posture sessions in which we modified the arm starting posture. The pre and post retracted posture sessions consisted of the same free-hand grasping task (18 trials) as those performed by Group 1 in Experiment 1, the object being located at 35 cm along the sagittal axis. The two retracted posture sessions (“Long-Distance” and “Short-Distance”), interleaved across the 18 participants, were composed of four blocks of 12 trials each (48 trials in total), during which participants, upon hearing an auditory go signal, were required to perform a reach and grasp movement without any tool (free-hand). During these retracted posture sessions, their initial hand position mimicked the one adopted in Experiment 1 for the tool use session (compare Figs 1c and 6b). The new starting position was set for each subject so that it would be equivalent to the one adopted if he/she had to use the tool. The micro-switch was repositioned underneath the participants’ hand (approximately 50 cm behind the starting point used in the pre and post sessions). In the “Long-distance” session, the object was in the same place as in Experiment 1, therefore at about 85 cm from the newly defined starting position. On the “Short-distance” session, the object was moved closer to the new starting position to maintain the same object distance as in the Experiment 1 and pre and post retracted posture sessions of Experiment 2 (see Fig. 6). The order of the “Short-Distance” and “Long-Distance” sessions was counterbalanced across participants. Each retracted posture session was followed by a post session in which participants went back to the same starting position adopted in the pre-retracted posture session. The entire experiment was video-recorded.

#### Kinematic recording and analysis

Kinematic recordings and analyses were as in Experiment 1. We excluded two participants from analyses: one because he systematically touched his left hand before grasping the object with his right hand, and one because he presented mean values exceeding 2.5 standard deviation of the mean population for at least two parameters. The data from the remaining 18 participants who took part in this within design (10 males; mean age ± SD: 22.2 ± 2.5; range from 18 to 26.6) were submitted to a full factorial design permutation analysis on pre and post session kinematic parameters with Order (“Short-distance reach” or “Long-distance reach” first) as a between-subject factor and Session (pre/post short-distance reach/post long-distance reach) as a within-subject factor to investigate (1) if the order of the retracted posture sessions had an influence on the kinematics, and (2) if the starting posture had an influence on the kinematics of free-hand movements. In addition to kinematics analysis, we reported the number of misreaches.

All data analyzed during this study are included in this published article (and its Supplementary Information files).

### Results

#### Dysmetric reaching

Following the repeated execution of reach to grasp movements performed with either scapular retraction posture, 5 out of 18 participants undershot the object in one or two trials at the beginning of the post session (4 participants after the long reach condition and 1 after the short reach one). Misreaching movements when going back to the starting-point were also observed, affecting 10 out of 18 participants (10 after the short-distance reach condition, and 4 after the long-distance reach one). By replicating the newly reported finding that people misreach objects when a retracted arm position is imposed during proprioceptively guided actions, we provide support to the hypothesis that misreaching can occur independently of tool use.

#### Movement kinematics

**Figure 8:**
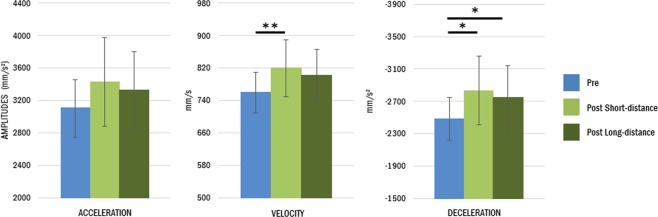
Movement kinematics following changes in arm posture. After both short- (light green) and long-distance sessions (dark green), participants showed higher peaks compared to before (blue). These changes are in the opposite direction to the ones induced by tool use in Experiment 1. Latencies (not shown) were not significantly modified. Asterisks denote significant differences between pre and post sessions. *<0.05; **<0.01; Bars illustrate mean values for each parameter and the 95% confidence interval.

Changing the hand starting posture affected some of the kinematic parameters of subsequent movements: noticeably, retracted arm posture increased the peak amplitudes (differently from tool-use, which decreased the same parameters). Permutations analyses revealed no main effect of Order (all ps > 0.153) neither interaction between Order and Session (all *p* > 0.13). Session had a significant effect on the wrist velocity peak (Fisher combination *K* = 9.71, *p* = 0.011, η²_p_ = 0.27), the deceleration peak (*K* = 8.17, *p* = 0.029, η²_p_ = 0.23), and the movement time (*K* = 7.69, *p* = 0.039, η²_p_ = 0.19) (see Fig. 8). Wrist velocity increased after the retracted arm posture in the “Short-distance reach” condition (pre: 760 vs post: 819 mm/s, t = 2.99, *p* = 0.007), a non-significant trend being visible for the “Long-distance reach” condition (pre: 760 vs post: 801 mm/s, t = 2.01, *p* = 0.054). A significant increase between pre and post sessions was observed for the deceleration peak (“Short-distance reach”: pre: −2485 vs post: −2833 mm/s², t = −2.52, *p* = 0.020; “Long-distance reach”: pre: −2485 vs post: −2756 mm/s², t = −2.08, *p* = 0.041), the two post sessions not differing from each other (t = 0.844, *p* = 0.416). As for the movement time, a significant decrease was observed only after the “Short-distance reach” (pre: 1318 vs post: 1219 ms, t = −2.34, p = 0.031; “long-distance reach”: pre: 1318 vs post: 1261 ms, t = −1.37, p = 0.184). When considering the latencies, retracted posture conditions had no significant effect on any of the parameters (acceleration: *K* = 2.14, *p* = 0.589; velocity: *K* = 0.931, *p* = 0.873; deceleration: *K* = 2.40, *p* = 0.523). Finally, the grip component of the movement was not affected by the retracted posture conditions (latency: *K* = 3.26, *p* = 0.351, aperture: *K* = 2.66, *p* = 0.467).

When grouping the parameters according to the movement component they describe, posture-dependent modifications did not reach significance for either the reaching (Fisher combination; *K* = 23.85, p = 0.080), or the grasping component (Fisher combination; *K* = 4.07, p = 0.395). Overall, and taking a very conservative stance, the results of Experiment 2 indicate that while misreaching behavior can be observed independent of tool-use, changing starting posture effects on kinematic are absent or, if anything, opposite to those observed following tool-use.

## Discussion

Can we incorporate a tool based on somatosensation alone? To answer this question, here we assessed whether healthy participants - relying solely on somatosensory inputs - could display the pattern of kinematic changes proposed to be the hallmark of tool incorporation in the unconscious body representation for action (see, for review^9^). Blindfolded healthy adults used a tool to grasp an object on a table. They were not provided with any visual information about either task specific features (e.g. object location, shape and size), or contextual features (e.g. working space or even the testing room). We only allowed participants to haptically explore both the object and the tool prior to the testing. Participants showed altered kinematics of reach-to-grasp movements after a brief period of proprioceptively-guided tool use. Movements performed after tool use exhibited significant changes in most of the transport component parameters, while grip parameters were not significantly affected. Such specificity of the kinematic modifications has been reported previously^32,45^ and have been interpreted as resulting from the absence of functional change in the grasping component. Indeed, actuating the tool in different planes (vertical vs horizontal squeezing), or even opposite direction (squeezing to open vs to close tool prongs) has not been considered critical for the achievement of the ultimate ‘function’ of the hand effector. For example, the kinematics of grasping is controlled independently of some of the biomechanical features of the grasping effector: by using a similar grabber to the one employed here (i.e., not enlarging the hand), Gentilucci and colleagues found striking kinematics similarities when comparing grasping an object with the tool and with the hand. Namely, the scaling of both peak velocity of aperture of the mechanical fingers and their maximal aperture varied as a function of object size just as did those of the biological fingers^51^. In tool-use trained monkeys, cortical motor neurons coding for hand grasping also fire while grasping with pliers (not enlarging the monkey hand), and this was true irrespective of whether normal pliers or reverse pliers (squeeze to open) were used to grasp an object^52^. However, one should observe kinematics changes at the level of the hand (grasping component) if some functional features of this effector are modified by a tool. Accordingly, we previously reported that the use of pliers or sticks - mimicking a functional elongation of the fingers – did induce modifications on the subsequent kinematic profile of the grasping component. Interestingly, in those conditions (i.e., using a tool that elongates the fingers but not the arm) the reaching component was not affected^45^. Similarly, in a tactile distance judgment task, Miller and colleagues reported that plastic modulation was observed on the hand after the use of a hand-shaped tool (making the hand functionally bigger), but not with an arm-shaped grabber (making the arm functionally longer). Conversely, modification of the arm representation was only observed after the use of the arm-shaped grabber, but not the hand-shaped one^19^. These studies have thus shown that body representation changes after tool-use are specific to the morpho-functional characteristics and the sensori-motor constrains imposed by the tool^9,19,45^. Importantly, these changes in kinematics were not spatially selective for the region where they used the tool (see^35^), thus ruling out the possibility that they represent a mere consequence of proprioceptive enhancement.

When considering the whole dataset of the current study (somatosensation only available, group 1&2), our conservative permutation-based approach made clear that somatosensation is sufficient for tool-use to modify both latencies and amplitude peaks of the transport phase. This pattern of kinematic changes is similar to that reported when participants used the same tool under visual guidance^5,34^. When statistically comparing part of the current data (somatosensation only available in group 1) with those from Cardinali and coworkers^5^ (somatosensation and vision available), it appeared that the multisensory condition may further enhance tool-induced effects on the peaks amplitude of the transport parameters. Moreover, Cardinali and colleagues^34^ reported that longer-armed participants tended to move with longer latencies and smaller peaks as compared to short-armed group. Here, correlation analyses confirmed the intimate relationship between arm length and transport kinematics: the longer the arm, the longer the latencies and the smaller the peaks amplitudes. This finding provides further support to the conclusion the pattern of kinematics changes observed here reflects the information that the arm to be moved is (represented as) longer after tool use.

In sharp contrast with the implicit arm representation for action, explicit arm representation seems immune to tool-use when only somatosensation is available. When participants were asked to explicitly estimate their arm length, we observed no effect of tool use, irrespective of whether the task was performed in the same or different direction/region of space with respect to where they used the tool. While tool use under visual guidance might lead to incorporation^53^, the present findings suggest that, although sufficient for implicit tool incorporation in the body representation for action, proprioception might not be sufficient to trigger tool incorporation at a more explicit level (see also^11,54^). While this finding could provide preliminary evidence that more explicit and conscious body representations, such as the Body Image, may be more resistant to some forms of tool use effects, further investigations using a wider range of Body Image tasks (see^55,56^) are needed to better delineate the conditions of permeability of the explicit body representation to tool incorporation.

This study critically extends our knowledge on the sensory information triggering tool embodiment and further confirms the specific kinematics fingerprints following tool-use. Here somatosensory tool-use revealed two major consequences: one is essentially kinesthetic and derives from tool-use per se (Exp. 1); a second one, statesthetic, derives from the modification in arm posture when wielding the tool at the starting position (i.e., scapular retraction, Exp. 2, see Fig. 6). While the former is reflected in the kinematics changes recalled above, the latter, newly observed here, took the form of dysmetric reaching movements (see supplementary for an example video). In a few trials right after tool-use, some participants misreached either the target object, or the home-pad on their way back to the starting point. Since changes in hand starting position are known to affect movements trajectories when no vision of the hand is allowed^47,50^, we took care of controlling also for this potential confound in experiment 2, by studying grasping movements performed -without tool- before and after a session in which hand posture was made to mimic the retracted one that participants experienced during tool use. As expected from changing arm posture, experiment 2 revealed the same kind of misreaching errors, that is misses of the object and/or the starting position. While this type of misreaching errors were observed after tool use in experiment 1, the control experiment 2 also makes clear that they cannot be attributed to the incorporation of the tool, as they were also present when the mere arm posture was modified without the tool. Experiment 2 also served the purpose of controlling for spurious test-retest effects of movement repetition and fatigue. At odds with the results obtained after tool use, this control experiment showed no significant change in kinematics of either the transport or the grasping component. If anything, participants tended to exhibit higher peaks in the transport kinematic parameters, instead of the lower peaks reported, here and elsewhere, following tool use^5^. Altogether, these findings clarify that separable effects can be observed and specifically attributed to either tool-incorporation, or changes in perceived hand posture.

Somatosensory signals evoked by tool use per se are thus sufficient for incorporating a tool that has never been seen, nor used, before. These findings complement a longitudinal study on a deafferented patient where no tool incorporation was observed due to the lack of proprioception^28^. By showing that a temporary absence of vision does not prevent tool incorporation in healthy participants, here we provide the lacking piece of evidence supporting the hypothesis that somatosensation is not only necessary^28^, but is also sufficient for tool incorporation. An interesting issue concerns the duration of the tool induced changes on body representation. By experimental design, we observed that it lasted during the whole duration of the post-session block, which is about 20 min depending on the order of the tasks. As we and others have proposed, this may be a transient effect and it would make adaptive sense if, similar to an elastic band, the arm representation would subsequently go back to its original size^57,58^ (for review^9^). As indeed pointed out by Longo and colleagues, plasticity might occur over multiple timescales dependent on usage frequency^59^. Indeed, long-lasting plasticity induced by tools, has been observed in the peripersonal space of blind people using their cane daily to navigate^60^, suggesting that some massive exercise might lead to longer lasting changes. This issue definitely deserves further investigation.

These findings also nicely parallel the previous observation that tool modifies peripersonal space even in the absence of vision^60^ as shown by blindfolded adults whose peripersonal space was limited to their hand, but was extended to include the tool after a few minutes of practice. Here we extend this knowledge, indicating that tool-use in blindfolded participants not only affects the space representation, but also the body representation for action. While somatosensation is sufficient, when available, vision certainly plays an important role in tool embodiment. Indeed, in a recent study, Miller and collaborators^17^ leveraged a visual illusion to investigate tool use effects. Participants saw the mirror reflection of their right arm grasping with a tool, thus illusorily experiencing tool-use in their left arm. After this tool-use illusion, tactile perception was modified in the stationary, unseen left arm. A control experiment additionally showed a quantitatively similar tactile effects could be obtained following actual tool use with the left arm. Vision thus can play an important role in determining tool-use effects. In the same vein, here we additionally provide a comparison with previous work that used the same paradigm under condition where both somatosensation and vision were available and found that vision may also enhance tool incorporation: while the full pattern of kinematic changes was present in either combination of sensory modalities, tool-use under vision and somatosensation reduced kinematics peaks amplitude even more than blindfolded tool-use (i.e., somatosensation alone). In this respect, we recently observed that tool-use imagery was sufficient to induce tool incorporation^33^. In this case, possibly according to the individual imagery style (visual and/or kinaesthesic), both proprioceptive and visual imagery of tool-use might have contributed to tool incorporation. This is also in line with studies on paralyzed patients, where mere tool-use observation was sufficient to induce plasticity on body representation^38^. While our findings demonstrate that a temporary loss of vision in not sufficient to prevent tool incorporation in the body representation for action, a thorough assessment of the role played by vision awaits for future investigations of people suffering from permanent visual loss. Serino and collaborators’ study^60^ cited above indeed illustrated that blind people displayed more permanent changes than blindfolded sighted participants in their peripersonal space representation. Quite interestingly, it seems that congenitally blind participants can remap objects in their peripersonal space, or in others’ peripersonal space as well as sighted participants^61^, reinforcing the need for further investigations.

The present study additionally examined, for the first time, the kinematics of movements performed in a direction different from that acted upon during tool use, and found that tool-induced kinematic changes did not interact with the specific direction/region of space in which the tool has been used. While some generalization effects of tool use have been previously reported (from reaching to pointing movements, see^5^), the present findings suggest that kinematics modifications may be arm-anchored, strongly supporting the idea that tool use affects the represented body morphology.

When discussing tool incorporation in the framework of motor control, it is important to assess the possible relationships between the effects we observed after tool-use and some of the most typical ‘aftereffects’ following sensorimotor learning (see for review^62^). For example, aftereffects following learning to walk with a weight (and thus higher torque) applied to a foot, would manifest as opposite to the effect induced by the weight itself, which lowers the maximum toe trajectory height. Comparing post-weight with pre-weight brings the toes being moved along a higher trajectory after release from weight^63^. In sharp contrast, the results reported here (and elsewhere) after grabber-use do not display shorter latencies and larger peaks than before tool-use, as one should expect from an ‘aftereffect’ perspective. Indeed, grasping objects with the same tool employed here has been shown to lengthen the latencies and reducing the peaks of the tool-transport component^5,34^. Contrasting with the direction of the effect which would be typically expected from most sensorimotor learning tasks (that is, shorter latencies and larger peaks), the direction of the effects reported here and in previous similar tool-use studies display the opposite pattern (that is, longer latencies and smaller peaks). Relatedly, transporting with the hand a heavy weight, participants likely applying a more important force to displace heavy (as compared to lighter) objects, was found to lengthen the latencies and reducing the amplitudes of transport parameters^64^. These kinematics changes are similar to those we observed during tool-use^5,34^. Importantly however, removing or reducing the weight typically induces higher amplitudes and shorter latencies with respect to the control (pre) condition, as there is no longer weight inertia to be exceeded by the applied force^65,66^. These results corroborate the ones by Lam and colleagues^63^ and contrast with the direction of the kinematics effects we have observed after tool-use both here, and in previous work. Thus, the known effects produced by release from weight and torque are not compatible with the lengthening of the latencies and the decrease of the amplitudes we consistently reported after tool-use. We therefore consider tool-use effects of the kind reported here differ from motor learning induced aftereffects and may help disambiguating between changes occurring at the level of the environment (aftereffects following motor learning of modified external conditions) from those occurring at the level of the state estimation of the body (modification of arm length representation following tool use). In this respect, it is important to consider tool use effects in the framework of internal models of action control (see^45,67^). To execute a movement, one has to anticipate and plan her action, as optimally as possible, through the inverse model of action, which is updated based on sensory signals. To avoid costly delays, sensory consequences of actions are predicted in the forward model, allowing for a better action tuning. As suggested elsewhere^45,67^, the body schema can be operationalized into a specific state estimate of the body, akin to hand position, or posture^68^. In agreement with recent work by Tang and collaborators^69^, the body schema would not contain the same motor program to be applied to either the arm or the tool, but would allow for the inverse model to be informed about a change in the state of the body. In other words, reaching with a tool would imply updating the state of the arm, i.e. the length parameter in the appropriate internal model. State estimation is a bridge between measured consequences of actions and update of inverse models, as well as between merely predicted consequences from forward model and update of inverse model^70^. Body representation plasticity leading to tool incorporation could thus originate from both measured and predicted feedbacks, according to the current availability of sensory information and the task’s specific constraints. Measured and predicted actions consequences may thus lead to tool-dependent kinematic changes that have been reported to follow, respectively, actual movements^5,17,19,45^ and imagined movements^33^. Richer sensory information leading to better-tuned models, the combination of vision and proprioception would logically lead to better tool incorporation as compared to proprioception or imagery alone.

In conclusion, here we report that somatosensory inputs (proprioceptive and haptic information) alone are sufficient for tool incorporation in healthy participants, suggesting an important role of somatosensation in the maintenance of the body representation for action.

## Supplementary information

Pre & Post tool-use session for a single subject (Exp 1, group 1)

Dataset 1

## References

[1] Iriki A, Tanaka M, Iwamura Y (1996). Coding of modified body schema during tool use by macaque postcentral neurones. Neuroreport.

[2] Maravita A, Spence C, Clarke K, Husain M, Driver J (2000). Vision and touch through the looking glass in a case of crossmodal extinction. Neuroreport.

[3] Berti A, Frassinetti F (2000). When far becomes near: remapping of space by tool use. J. Cogn. Neurosci..

[4] Farnè A, Làdavas E (2000). Dynamic size-change of hand peripersonal space following tool use. Neuroreport.

[5] Cardinali L (2009). Tool-use induces morphological updating of the body schema. Curr. Biol..

[6] Sposito A, Bolognini N, Vallar G, Maravita A (2012). Extension of perceived arm length following tool-use: clues to plasticity of body metrics. Neuropsychologia.

[7] Canzoneri E (2013). Tool-use reshapes the boundaries of body and peripersonal space representations. Exp. Brain Res..

[8] Kilteni K, Ehrsson HH (2017). Sensorimotor predictions and tool use: Hand-held tools attenuate self-touch. Cognition.

[9] Martel M, Cardinali L, Roy AC, Farnè A (2016). Tool-use: An open window into body representation and its plasticity. Cogn. Neuropsychol..

[10] de Vignemont F (2010). Body schema and body image–pros and cons. Neuropsychologia.

[11] Kammers MPM, de Vignemont F, Verhagen L, Dijkerman HC (2009). The rubber hand illusion in action. Neuropsychologia.

[12] Rossetti A, Romano D, Bolognini N, Maravita A (2015). Dynamic expansion of alert responses to incoming painful stimuli following tool use. Neuropsychologia.

[13] Patané, I., Iachini, T., Farnè, A. & Frassinetti, F. Disentangling Action from Social Space: Tool-Use Differently Shapes the Space around Us. *PLoS One* **11** (2016). 10.1371/journal.pone.0154247 PMC4856400 27144720

[14] Maravita A, Husain M, Clarke K, Driver J (2001). Reaching with a tool extends visual-tactile interactions into far space: evidence from cross-modal extinction. Neuropsychologia.

[15] Serino A, Canzoneri E, Avenanti A (2011). Fronto-parietal areas necessary for a multisensory representation of peripersonal space in humans: an rTMS study. J. Cogn. Neurosci..

[16] Cléry J, Guipponi O, Wardak C, Ben Hamed S (2015). Neuronal bases of peripersonal and extrapersonal spaces, their plasticity and their dynamics: knowns and unknowns. Neuropsychologia.

[17] Miller LE, Longo MR, Saygin AP (2017). Visual illusion of tool use recalibrates tactile perception. Cognition.

[18] Costantini M, Ambrosini E, Sinigaglia C, Gallese V (2011). Tool-use observation makes far objects ready-to-hand. Neuropsychologia.

[19] Miller LE, Longo MR, Saygin AP (2014). Tool morphology constrains the effects of tool use on body representations. J. Exp. Psychol. Hum. Percept. Perform..

[20] Miller LE (2018). Sensing with tools extends somatosensory processing beyond the body. Nature.

[21] Takahashi C, Watt SJ (2017). Optimal visual-haptic integration with articulated tools. Exp. Brain Res..

[22] Clifton RK, Rochat P, Robin DJ, Berthier NE (1994). Multimodal perception in the control of infant reaching. J. Exp. Psychol. Hum. Percept. Perform..

[23] Clifton RK, Muir DW, Ashmead DH, Clarkson MG (1993). Is visually guided reaching in early infancy a myth?. Child Dev..

[24] McCarty ME, Clifton RK, Ashmead DH, Lee P, Goubet N (2001). How infants use vision for grasping objects. Child Dev..

[25] Head H, Holmes G (1911). Sensory disturbances from cerebral lesions. Brain.

[26] Parsons LM (1987). Imagined spatial transformations of one’s hands and feet. Cognit. Psychol..

[27] Shenton JT, Schwoebel J, Coslett HB (2004). Mental motor imagery and the body schema: evidence for proprioceptive dominance. Neurosci. Lett..

[28] Cardinali L, Brozzoli C, Luauté J, Roy AC, Farnè A (2016). Proprioception is necessary for Body Schema plasticity: evidence from a deafferented patient. Front. Hum. Neurosci..

[29] Fitzpatrick P, Carello C, Turvey MT (1994). Eigenvalues of the inertia tensor and exteroception by the ‘muscular sense’. Neuroscience.

[30] Jacobs S, Bussel B, Combeaud M, Roby-Brami A (2009). The use of a tool requires its incorporation into the movement: evidence from stick-pointing in apraxia. Cortex J. Devoted Study Nerv. Syst. Behav..

[31] Michaels CF, Isenhower RW (2011). Information space is action space: perceiving the partial lengths of rods rotated on an axle. Atten. Percept. Psychophys..

[32] Cardinali L, Brozzoli C, Farnè A (2009). Peripersonal space and body schema: two labels for the same concept?. Brain Topogr..

[33] Baccarini M (2014). Tool use imagery triggers tool incorporation in the body schema. Front. Psychol..

[34] Cardinali L (2012). Grab an object with a tool and change your body: tool-use-dependent changes of body representation for action. Exp. Brain Res..

[35] Wong JD, Wilson ET, Gribble PL (2011). Spatially selective enhancement of proprioceptive acuity following motor learning. J. Neurophysiol..

[36] Declaration of Helsinki: Ethical principles for medical research involving human subjects. **310**, 2191–2194, 10.1001/jama.2013.281053. *J. Am. Med. Assoc*. (2013). 10.1001/jama.2013.281053 24141714

[37] World Medical Association. World Medical Association. (2013).

[38] Garbarini, F. *et al*. When your arm becomes mine: Pathological embodiment of alien limbs using tools modulates own body representation. *Neuropsychologia*. 10.1016/j.neuropsychologia.2014.11.008 (2014). 10.1016/j.neuropsychologia.2014.11.008 25448852

[39] Longo MR (2015). Implicit and explicit body representations. Eur. Psychol..

[40] Longo MR, Haggard P (2012). Implicit body representations and the conscious body image. Acta Psychol. (Amst.).

[41] Basso D, Finos L (2012). Exact Multivariate Permutation Tests for Fixed Effects in Mixed-Models. Commun. Stat. - Theory Methods.

[42] Finos L, Basso D (2014). Permutation Tests for Between-unit Fixed Effects in Multivariate Generalized Linear Mixed Models. Stat. Comput..

[43] Finos, L. Livio Finos, with contributions by Corrado Lanera, Florian Klinglmueller, Dario Basso, Aldo Solari, Lucia Benetazzo, Jelle Goeman and Marco Rinaldo. flip: Multivariate Permutation Tests. R package version 2.4.3.0021, https://CRAN.R-project.org/package = flip. (2015).

[44] R Core Team. R: A language and environment for statistical computing. R Foundation for Statistical Computing, Vienna, Austria. https://www.R-project.org/ (2018).

[45] Cardinali L, Brozzoli C, Finos L, Roy AC, Farnè A (2016). The rules of tool incorporation: Tool morpho-functional & sensori-motor constraints. Cognition.

[46] Pesarin, F. Multivariate Permutation Tests: With Applications in Biostatistics. (Wiley 2001).

[47] Desmurget M, Gréa H, Prablanc C (1998). Final posture of the upper limb depends on the initial position of the hand during prehension movements. Exp. Brain Res..

[48] Ghilardi MF, Gordon J, Ghez C (1995). Learning a visuomotor transformation in a local area of work space produces directional biases in other areas. J. Neurophysiol..

[49] Kritikos A, Jackson GM, Jackson SR (1998). The influence of initial hand posture on the expression of prehension parameters. Exp. Brain Res..

[50] Vindras P, Desmurget M, Prablanc C, Viviani P (1998). Pointing errors reflect biases in the perception of the initial hand position. J. Neurophysiol..

[51] Gentilucci M, Roy AC, Stefanini S (2004). Grasping an object naturally or with a tool: are these tasks guided by a common motor representation?. Exp. Brain Res..

[52] Umiltà MA (2008). When pliers become fingers in the monkey motor system. Proc. Natl. Acad. Sci. USA.

[53] Miller, L. E., Longo, M. R. & Saygin, A. P. Tool use modulates both conscious and unconscious representations of body shape. Presented at ASSC 2013 in San Diego, C. A. in (2013).

[54] Cardinali L (2011). When action is not enough: tool-use reveals tactile-dependent access to Body Schema. Neuropsychologia.

[55] Perera AT, Newport R, McKenzie KJ (2017). Changing hands: persistent alterations to body image following brief exposure to multisensory distortions. Exp. Brain Res..

[56] Stone KD, Keizer A, Dijkerman HC (2018). The influence of vision, touch, and proprioception on body representation of the lower limbs. Acta Psychol. (Amst.).

[57] de Vignemont F, Farnè A (2010). Widening the body to rubber hands and tools: what’s the difference?. Rev Neuropsychol.

[58] de Vignemont F (2011). Embodiment, ownership and disownership. Conscious. Cogn..

[59] Longo MR, Sadibolova R, Tamè L (2016). Embodying prostheses - how to let the body welcome assistive devices: Comment on ‘The embodiment of assistive devices-from wheelchair to exoskeleton’ by M. Pazzaglia and M. Molinari. Phys. Life Rev..

[60] Serino A, Bassolino M, Farnè A, Làdavas E (2007). Extended multisensory space in blind cane users. Psychol. Sci..

[61] Ricciardi E (2017). Peripersonal space representation develops independently from visual experience. Sci. Rep..

[62] Krakauer JW, Mazzoni P (2011). Human sensorimotor learning: adaptation, skill, and beyond. Curr. Opin. Neurobiol..

[63] Lam T, Wolstenholme C, Yang JF (2003). How do infants adapt to loading of the limb during the swing phase of stepping?. J. Neurophysiol..

[64] Roy AC (2013). Syntax at hand: common syntactic structures for actions and language. PloS One.

[65] Johansson RS, Westling G (1988). Coordinated isometric muscle commands adequately and erroneously programmed for the weight during lifting task with precision grip. Exp. Brain Res..

[66] Gordon AM, Forssberg H, Johansson RS, Westling G (1991). The integration of haptically acquired size information in the programming of precision grip. Exp. Brain Res..

[67] Martel, M., Cardinali, L., Roy, A. C. & Farnè, A. Tool use unravels body morphology representation in the brain. in *F. de Vignemont and A. Alsmith (eds) The subject’s matter: the body and self-awareness, MIT Press* (2017).

[68] Wolpert DM, Ghahramani Z (2000). Computational principles of movement neuroscience. Nat. Neurosci..

[69] Tang R, Whitwell RL, Goodale MA (2016). Unusual hand postures but not familiar tools show motor equivalence with precision grasping. Cognition.

[70] Shadmehr R, Krakauer JW (2008). A computational neuroanatomy for motor control. Exp. Brain Res..

